# Transcriptome and Metabolome Analysis of a Late-Senescent Vegetable Soybean during Seed Development Provides New Insights into Degradation of Chlorophyll

**DOI:** 10.3390/antiox11122480

**Published:** 2022-12-16

**Authors:** Jian Wang, Guang Chen, Xuetong Li, Xujun Fu, Sujuan Li, Xiaoyuan Tao, Zhong-Hua Chen, Shengchun Xu

**Affiliations:** 1 Central Laboratory, State Key Laboratory for Managing Biotic and Chemical Threats to the Quality and Safety of Agro-Products, Zhejiang Academy of Agricultural Sciences, Hangzhou 310021, China; 2 Hangzhou National Sub-Center of Soybean Improvement, Institute of Crop and Nuclear Technology Utilization, Zhejiang Academy of Agricultural Sciences, Hangzhou 310021, China; 3 School of Science, Western Sydney University, Penrith, NSW 2751, Australia; 4 Hawkesbury Institute for the Environment, Western Sydney University, Penrith, NSW 2751, Australia

**Keywords:** senescence, soybean seed, late-senescent, chlorophyll metabolism, flavonoids

## Abstract

(1) Background: Senescence represents the final stage of plant growth and development, which transfers nutrients to growing seeds and directly affects the yield and quality of crops. However, little is known about chlorophyll degradation in developing and maturing seeds, in contrast to leaf senescence; (2) Methods: RNA-Seq was used to analyze the differentially expressed genes of different late-senescent germplasms. A widely untargeted metabolic analysis was used to analyze differential metabolites. In addition, qRT-PCR was conducted to detect gene expression levels; (3) Results: Transcriptome analysis revealed that ZX12 seeds have a higher expression level of the chlorophyll synthesis genes in the early stage of maturity, compared with ZX4, and have a lower expression level of chlorophyll degradation genes in the late stage of maturity. Flavonoids were the primary differential metabolites, and ZX12 contains the unique and highest expression of three types of metabolites, including farrerol-7-O-glucoside, cyanidin-3-o-(6′-o-feruloyl) glucoside, and kaempferide-3-o-(6′-malonyl) glucoside. Among them, farrerol-7-O-glucoside and cyanidin-3-o-(6′-o-feruloyl) glucoside are flavonoid derivatives containing mono and dihydroxy-B-ring chemical structures, respectively; and (4) Conclusions: It is speculated that the two metabolites can slow down the degradation process of chlorophyll by scavenging oxygen-free radicals in the chloroplast.

## 1. Introduction

Vegetable soybeans (edamame) are harvested at an immature stage, i.e., R6 (full seed stage) versus R8 (full maturity stage), and feature a large, sweet, nutty, and mild-flavored seed [1,2]. Continuous improvement in understanding nutritional properties and transformation to healthier lifestyles have allowed vegetable soybean to flourish in recent decades [3]. Nutrient composition analysis has shown that vegetable soybean has a higher nutrient composition than green peas (*Pisum sativum* L.), including higher protein, calcium, phosphorus, potassium [4], and ascorbic acid contents. In addition, vegetable soybean contains 50% more isoflavones than mature soybean seeds [5,6], of which malonylglycoside is the most abundant form. Vegetable soybean is also one of the few plant-based foods to contain essential amino acids such as lysine and tryptophan. It is also high in sugar and minerals, making it an excellent source of secondary metabolites [7]. In terms of physical characteristics, the appearance of fresh pods and seeds is very important, and the degree of greenness in pods and seeds is an essential commercial characteristic [2,8]. Late-senescent plants are beneficial for prolonging photosynthetic capacity during the whole growth period, and improving the synthesis and transportation of dry matter, which is closely related to crop yield [9,10,11]. Moreover, delaying the senescence of vegetable soybean seeds can inhibit the deterioration of sugar, amino acids, and ascorbic acid in harvesting, storage, transportation, and processing. It can prolong the picking period to relieve labor and support long-distance transportation and extended storage [12,13].

Plant senescence is a natural developmental process at the end of plant development. During plant senescence, chlorophyll (Chl) and other large molecules are degraded, the photosynthetic capacity of leaves is reduced, and nutrients in the aging tissues are transported to young tissues and reproductive organs [14,15,16]. Senescence is induced by growth stage and environmental stress, such as darkness, drought, nutrient deficiency, high salt, low temperature, ozone, and pathogen infection. These processes are often accompanied by the accumulation of reactive oxygen species (ROS) and decreased activity of antioxidant enzymes (SOD, CAT, and APX) in cells [17,18]. Senescence is also a maturation process, which is crucial for the life cycle of the plant and can directly affect the production capacity of crops [19]. For some crop plants, chlorophyll synthesis and degradation also exist in seeds during maturation [20]. At the seed maturation stage, Chl also has photosynthetic capacity, but the carbon assimilation ability of seed Chl is relatively weak compared with that in leaves [21]. Therefore, a portion of the organic compounds required for seed development can be synthesized from seeds. At the late stage of seed maturity, the seed must go through a dormancy period, accompanied by dehydration and Chl degradation, to complete its post-maturation development. However, residual Chl can still be detected in the mature seed tissues of many plant species [22]. The apparent reason for this finding is that the Chl degradation pathway is disturbed during the final maturation step. Notably, degradation of Chl during senescence also leads to a decline in plant photosynthetic capacity, hence its carbon assimilation capacity. Therefore, the delay of seed senescence will increase the amount of fixed carbon that can be used for seed filling, which is directly related to increased crop yield [17,23].

The term “stay green” is occasionally used as a synonym to describe the senescence process, strictly referring to a mutant with impaired chlorophyll metabolism [24]. The term “stay green” is not widely used in soybeans, but it is often used in other crops such as rice, corn, sorghum, etc. [25]. Functional and cosmetic stay-green traits can be divided into five types (Thomas and Howarth, 2000): Type A, senescence is initiated late but proceeds normally. Type B, initiates senescence at the same time as wild types, but the degradation of Chl is significantly slower. Type C, Part of the Chl content can be maintained, but the senescence process proceeds normally, and the photosynthetic capacity is impaired. Type D, plants die from extreme damage (freezing, drying, etc.), and dead leaves remain green. Type E, retains high chlorophyll content without increased photosynthesis. It is functional for type A and type B to stay-green because the photosynthetic activity is prolonged during seed maturation. The C, D, and E types that stay-green are cosmetics; Although chlorophyll was retained, photosynthetic activity was similar to that of the wild type at the same senescence stage [10]. Most described stay-green mutants are cosmetic mutants resulting from impaired chlorophyll metabolism [26,27,28,29,30,31,32]. In soybean, three genotypes of stay-green mutants controlled by nuclear genes (*G* and *d1d2*) and a cytoplasmic gene (*cytG*) belonging to type C were found in leaves and cotyledons. It was found that either a dominant (*G*) gene or a pair of recessive (*d1d2*) genes controlled the two stay-green traits of Mendelian inheritance [33,34,35,36]. *G* alone controls the stay-green phenotype of seed coats, while *d1d2* controls the green retention traits of the seed coat, cotyledon, pod, and vegetative portions [37]. The *Gd1d2* exhibits the stay-green trait of all tissues. The *cytG* mutant exhibits the stay-green phenotype in leaves, pods, seed coats, and cotyledons [38,39]. Soybean *GmSGR1* and *GmSGR2* are homologous candidates of *D1* and *D2*, and the loss-functional transgenic lines of *GmSGR1* and *GmSGR2* show the same stay-green phenotype as *d1d2* mutant [40,41]. However, the underlying mechanism by which *SGR* and Chl catabolic enzymes (*NYC1*, *HCAR*, *PPH*, *PaO*, etc.) regulate Chl degradation in green seeds is still unclear [42,43]. To isolate *cytG*, the soybean chloroplast genome was extensively sequenced. It was found that *psbM*, a small subunit encoding photosystem II, may co-regulate leaf chlorophyll degradation with chlorophyll b reductase (*NYC1*) [44]. 

Delayed senescence can improve vegetable soybean yield, resistance, appearance quality, storage, transportation properties, and other aspects. It directly affects the production and commercial characteristics of vegetable soybeans [9,10,39,40]. In this study, we first identified two vegetable soybean germplasms with different delayed senescence characteristics (wild-type Zhexian No. 4 and late-senescent type Zhexian No. 12). We examined their phenotypic characteristics and revealed ZX12 had a soybean seed delayed senescence phenotype. We further sequenced the transcriptomes of the two germplasms at a range of time points (days after flowering, DAF) and performed KEGG enrichment analysis on differentially expressed genes. The results highlighted key genes of Chl metabolism and the photosynthetic pathway among differentially expressed genes, at different DAF. We also performed untargeted metabolome analysis on seeds of the two germplasms collected at different DAF; this analysis revealed that flavonoids were the primary differentially abundant metabolites. Correlation analysis of the differentially expressed genes and flavonoid and anthocyanin metabolites revealed that expression was positively correlated with their metabolites for most key genes of flavonoid metabolism, indicating that flavonoids play a key role in the soybean seed late-senescent phenotype. Therefore, this study helps elucidate the mechanism by which the delayed senescence trait is regulated during the senescence process of vegetable soybean seeds, and provides us with novel insights into avenues of crop genetic improvement.

## 2. Materials and Methods

### 2.1. Plant Materials and Sample Collection

Samples were collected from two soybean germplasms, Glycine max cv. ‘Zhexian No. 4’ (hereafter referred to as ZX4) and Glycine max cv. ‘Zhexian No. 12’ (hereafter referred to as ZX12), both grown in the breeding field of the Zhejiang Academy of Agricultural Sciences, Hangzhou, China. A randomized block design with three replications was used. For each variety, 18 pods with the same growth potential were collected at each of the seven-time points: 20, 25, 30, 35, 40, 45, and 50 days after flowering (DAF). Half of the samples were used to determine pod and seed weight, Chl content, and Chl fluorescence intensity; the other half were immediately frozen in liquid nitrogen and stored at −80 °C for subsequent starch, sugar, and antioxidant enzyme activity analyses, along with RNA extraction.

### 2.2. Measurement of Chl Content and Fluorescence Intensity

Seeds were soaked in 95% ethanol to extract the Chl. The extract was centrifuged at 5300× *g* for 10 min, after which the absorbance of the supernatant was read at 649 nm and 665 nm. Total Chl, Chl a, and Chl b contents were then calculated according to the respective equations C_a_ = 13.36A665 − 5.19A649, C_b_ = 27.43A649 − 8.12A665, and C_a+b_ = 5.24A665 + 22.24A649 [45]. To measure Chl auto-fluorescence intensity in the seed epidermis, the epidermis was sliced to 50 μM thickness with a frozen microtome and made into temporary mount slides. The slides were then observed under a laser scanning confocal microscope (Zeiss, Oberkochen, Germany) and excited at 488 nm. The detection range for excitation light was 620–730 nm, and fluorescence intensity was measured with the ZEN blue software [46].

### 2.3. Pod Moisture Content, Seed Moisture Content, and Seed Dry Weight Determination

Pod and seed moisture content was measured using the high constant temperature oven dry method [47]. About 5–8 g of soybean seeds were placed in an aluminum dish and dried in an oven at 130 °C for two hours until a constant weight was achieved. Then the sample was weighed, and the moisture content was calculated as follows: Moisture content (%) = (W − W1) × 100/W, where W is the weight of the seed or pod before drying and W1 is the weight after drying. One hundred-seed dry weights were dried and determined in the same manner. 

### 2.4. Soluble Sugars, Starch, and Antioxidant Enzyme Activity Determinations

Total soluble sugars (TSS) were quantified in 80% ethanol extracts of seed tissues. First, a sample of 0.5 g of seeds was crushed in 5 mL of 80% (*v*/*v*) ethanol. All soluble fractions were centrifuged at 3500× *g* for 10 min. The supernatants were collected and stored at 4 °C for TSS determination. TSS was assessed by reacting 0.1 mL of alcoholic extract with 3 mL freshly prepared anthrone (150 mg anthrone + 100 mL 72% [*w*/*w*] H_2_SO_4_) and placing it in a boiling water bath for 10 min. After cooling, the absorbance at 625 nm was determined in a Bausch and Lomb 2000 Spectronic spectrophotometer [48]. Starch content was determined by the modified iodine chromogenic method [49]. To determine the activities of superoxide dismutase (SOD), peroxidase (POD), and catalase (CAT). A total superoxide dismutase (T-SOD) assay kit (Jiancheng, China), peroxidase assay kit (Jiancheng, China) and catalase assay kit (Jiancheng, China) were used following the user manuals. 

### 2.5. Stranded-RNA-Seq Library Construction and Sequencing

Total RNAs of seeds were extracted from samples of 20 d, 30 d, 40 d, and 50 d after flowering from both germplasms. Samples were prepared in eight groups, with three samples per event for 24 libraries. rRNA was removed from the sample using Ribo-Zero rRNA Removal Kits (Epicentre, Madison, WI, USA). A fragmentation buffer was then used to generate short rRNA-depleted RNA fragments of ~250 bp, which were then used as templates. Random hexamers were used to synthesize the first-strand cDNA. Buffer, dATP, dUTP, dCTP, dGTP, DNA polymerase I and RNase H were added to synthesize the second cDNA strand. AMPure XP beads (Beckman Coulter, Miami, FL, USA) were used to purify the double-strand cDNA, which was then end-repaired, poly-A tailed, adaptor-ligated, and size selected. The strand with uracil was then degraded, and the target strand was PCR enriched. Qubit 2.0 Fluorometer (Life Technologies, Carlsbad, CA, USA) and Agilent 2100 Bioanalyzer (Agilent, Palo Alto, CA, USA) were used to verify the quality of the sequencing library. The library products were then sequenced on an Illumina HiSeq X-Ten system. Quality control analysis on the resulting fastq sequencing files was performed using FastQC (Babraham Bioinformatics, Cambridgeshire, UK).

### 2.6. Read Mapping and Quantifying

CLC Genomics Workbench (QIAGEN, https://www.qiagenbioinformatics.com/products/clc-genomics-workbench/ (accessed on 10 April 2021)) is a software allowing for comprehensive analysis of RNA-seq data [50]. Quality control analysis on the resulting fastq sequencing files was performed using “QC for Sequencing Reads” in the software. “Trim Reads” function was used to perform adaptor trimming. Clean reads were then mapped to the *Glycine max* reference genome (ftp://ftp.ensemblgenomes.org/pub/release-42/plants/embl/glycine_max/ (accessed on 12 April 2021)) using “RNA-Seq Analysis.” RPKM was used to measure the gene expression levels. The reproducibility of the experiment was measured using “PCA for RNA-Seq.” The above steps were performed using the software’s default parameters (https://resources.qiagenbioinformatics.com/manuals/clcgenomicsworkbench/current/User_Manual.pdf (accessed on 8 April 2021)). Differentially expressed genes (DEG) of the same period between the two germplasms were also detected using the implemented method in the CLC Genomics Workbench, while using ZX4 as the control. The FDR-corrected *p*-value ≥ 0.05 and |Log_2_(FC)| ≥ 1 were used as the screening criteria. DEGs analysis for the two germplasms across all time series was also performed using the same software and criteria.

### 2.7. Annotation of Differentially Expressed Genes

Gene Ontology (GO) annotations of *Glycine max* gene products were obtained from the Gene Ontology database (http://geneontology.org (accessed on 15 April 2021)). DEGs were evaluated for enrichment of terms in the Plant GO Slim. Terms were considered significantly enriched if the hypergeometric test implemented in GO-TermFinder 0.86 yielded a Bonferroni corrected *p*-value of less than 0.05 [51].

### 2.8. Real-Time Quantitative PCR Analysis

Total RNA was isolated from 20 and 40 DAF seeds using the EZNA Plant RNA Kit (Omega Bio-Tek, Norcross, GA, USA) following the manufacturer’s instructions. RNA integrity was verified with 1% agar gel electrophoresis and RNA concentration was measured using the BioDrop uLite (BioDrop, Cambridge, UK). The first cDNA strand was synthesized from 1 μg of total RNA using MonScript™ RTIII All-in-One Mix with dsDNase (Monad, Wuhan, China), according to the manufacturer’s instructions. Real-time quantitative RT-PCR was conducted in a Bio-rad CFX96™ (Bio-rad, Hercules, CA, USA) using MonAmp™ SYBR^®^ Green qPCR Mix (None ROX) (Monad, Wuhan, China). The 15 μL reaction mixture contained 2 μL of a diluted template (10 μL of the generated first-strand cDNA diluted with 90 μL ddH_2_O), 7.5 μL of MonAmp™ SYBR^®^ Green qPCR Mix, 0.4 μL of each of the two gene-specific primers (10 μM), and 4.7 μL ddH_2_O. The thermocycler program was as follows: 95 °C for 30 s, then 40 cycles of 95 °C for 10 s, 60 °C for 15 s, and 72 °C for 20 s. A melting curve analysis was conducted following each assay to confirm the specificity of the amplicon for each primer pair. Gene-specific primers were designed using Primer 3 [52]. Relative gene expression values were calculated using the 2^−ΔΔ^CT method with the soybean gene *GmEF1b* as the reference [53]. The gene-specific primers are listed in Supplemental Table S1.

### 2.9. Metabolic Analysis

A widely untargeted metabolic analysis was performed on ZX4 and ZX12 seeds collected at 20, 30, and 40 DAF, with three biological replicates. Methanol and acetonitrile of chromatographic grade were purchased from Merck (GER), and standards were obtained from BioBioPha (BioBioPha, Kunming, China) and Sigma-Aldrich (Sigma-Aldrich, St. Louis, MO, USA). Samples to be analyzed were freeze-dried with a vacuum freeze dryer and ground into powder using a grinder (30 Hz, 1.5 min). Then, 100 mg of sample was dissolved in 1.2 mL 70% MeOH, and incubated, with vortexing every 30 min, for a total of six rounds of vortexing. Afterwards, samples were placed in the refrigerator at 4 °C overnight. The next day, the samples were centrifuged at 12,000 rpm for 10 min and the supernatant was extracted and filtered by a micropore filter (0.22 μM pore size). The samples were then transferred into sample bottles for subsequent UPLC-MS/MS analysis.

Ultra-performance liquid chromatography (SHIMADZU Nexera X2, https://www.shimadzu.com.cn/ (accessed on 30 May 2021)) coupled with tandem mass spectrometry (Applied Biosystems 4500 QTRAP, http://www.appliedbiosystems.com (accessed on 30 May 2021)) were used to perform the widely untargeted metabolic analysis of samples. Chromatographic separation was performed on an Agilent SB-C18 column (1.8 µm, 2.1 mm × 100 mm). The mobile phases consisted of ultrapure water with 0.1% formic acid (A) and acetonitrile with 0.1% formic acid (B). The gradient elution program was set as follows: 95:5 A/B at 0 min, 5:95 A/B at 9 min, 5:95 A/B at 10 min, 95:5 A/B at 11.10 min, and 95:5 A/B at 14 min. The flow rate was 0.35 mL/min, the column temperature was 40 °C, and the injection volume was 4 μL. The linear ion trap and QQQ scanning were performed using an AB4500 Q TRAP MS/MS system. MS acquisition utilized both positive and negative ion modes. The parameters for electrospray ionization (ESI) were as follows: source temperature, 550 °C; ion spray voltage, 5500 V (+)/−4500 V (−). QQQ scanning employed multiple reaction monitoring. 

Structural annotation of mass spectra was carried out using an in-house library (MetWare database). Quantitative analysis of metabolite features was performed by the mass spectrometer, in the course of multiple reaction monitoring. The Analyst 1.6.3 software was used to analyze mass spectrometry data, while MultiaQuant was used to calculate and correct each metabolite peak area. 

A blend of all samples was used as the quality control sample. Quality control samples were submitted to the analytical instrument once every ten samples to monitor the repeatability of the mass spectrometry analysis.

### 2.10. Data Availability

Sequence files supporting the results of this article are available from the NCBI Sequence Read Archive under the accession number (https://www.ncbi.nlm.nih.gov/bioproject/?term=PRJNA904140 (accessed on 22 November 2022), Accession: PRJNA904140). Metabolomics data used in the study can be viewed and accessed in The National Omics Data Encyclopedia (https://www.biosino.org/node/project/detail/OEP003774 (accessed on 28 November 2022), Project ID: OEP003774).

### 2.11. Statistic Analysis

Student’s *t*-tests were used to determine the significance levels of the Chl degradation index for ZX4 and ZX12. Significance levels: 0.01 < * *p* ≤ 0.05, 0.001 < ** *p* ≤ 0.01, *** *p* ≤ 0.001. Significant differences in Chl content (Chl a, Chl b and total Chl content), fluorescence intensity, and physiological characteristics (SOD, POD, and T-AOC activities) of ZX4 and ZX12 seeds, at different stages of maturity as indicated by the 1/2 LSD value, were calculated using SAS 9.0 (SAS Institute Inc., Cary, NC, USA). The relative transcript expression levels of genes were log2 transformed for analysis. Data clustering analysis and the quantitative color scheme were applied using Amazing Heatmap (https://github.com/CJ-Chen/TBtools (accessed on 12 July 2021)). Bar charts illustrating the relative expression levels of key factors involved in Chl synthesis, cycling, and degradation were generated with SigmaPlot version 12.5 (Systat Software, Inc., San Jose, CA, USA). Differences in gene expression levels between the ZX4 and ZX12 were evaluated for significance using analysis of variance, according to the general linear model procedure of SAS 9.0 (SAS Institute Inc., Cary, NC, USA). Differences between means were assessed by Fisher’s protected least significance difference (LSD) test at the 0.05 probability level. Orthogonal partial least squares discriminant analysis (OPLS-DA) was carried out using the MetaboAnalystR package in the R (v4.1.0) programming environment. C-Means clustering of metabolic abundance data was likewise carried out with the TCseq package.

## 3. Results

### 3.1. ZX12 Seeds Showed Higher Chl Content and Lower Chl Degradation Rate When Compared with ZX4

**Figure 1:**
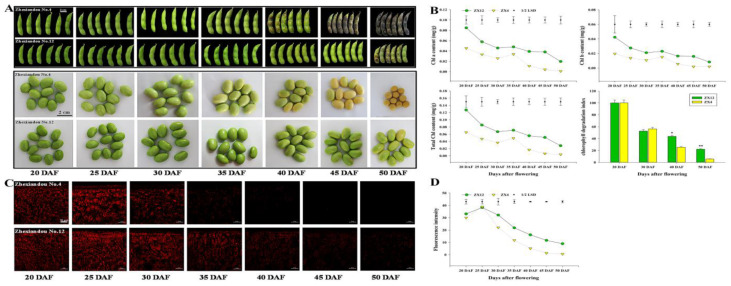
Chl content of soybean varieties ZX4 and ZX12. (**A**) Photographs of the phenotype of pods and seeds at different days (20 d, 25 d, 30 d, 35 d, 40 d, 45 d, and 50 d) after flowering. (**B**) Chl content (Chl a, Chl b, total Chl, and Rate of Chl degradation) of ZX4 and ZX12 seeds at different DAF. (**C**) Photographs of Chl autofluorescence intensity of ZX4 and ZX12 seeds at different DAF. (**D**) Chl fluorescence intensity of ZX4 and ZX12 seeds.

To identify regulatory factors related to the late-senescent phenotype in soybean seeds, we selected two germplasms, ZX4 (Zhexian No. 4) and ZX12 (Zhexian No. 12), which exhibit different Chl levels in daily production. In observing the phenotypes of pods and seeds, yellow spots appeared on the pods of ZX4 at 40 DAF, but for ZX12 did not appear until 50 DAF (Figure 1A). Measurements of Chl a, Chl b, and total Chl content revealed ZX12 seeds at 20 DAF to have significantly higher values than the seeds of ZX4 (Figure 1B). For both germplasms, Chl content in seeds decreased as DAF increased. No significant difference in the Chl degradation index was evident between the two germplasms at 20 DAF and 30 DAF. However, at 40 and 50 DAF, ZX12 had index values significantly higher than ZX4 (Figure 1B). This finding indicates that ZX12 has lower Chl degradation efficiency than ZX4. Chl auto-fluorescence assays of the soybean seed epidermis showed that Chl fluorescence intensity also decreased as DAF increased, and the fluorescence intensity of ZX12 was higher than that of ZX4 after 25 DAF (Figure 1C,D).

### 3.2. Physiological Character of ZX4 and ZX12 Seeds at Different Stages of Maturity

**Figure 2:**
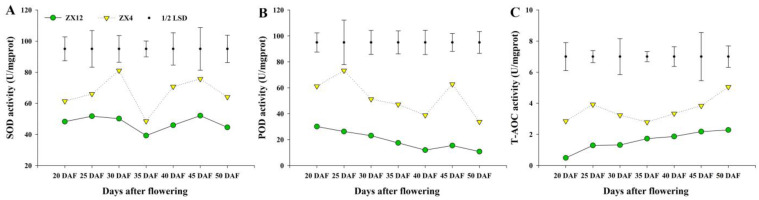
SOD, POD, and T-AOC activities of ZX4 and ZX12 seeds at different maturity stages. (**A**) SOD activities of ZX4 and ZX12 seeds at different maturity stages. (**B**) POD activities of ZX4 and ZX12 seeds at different maturity stages. (**C**) T-AOC activities of ZX4 and ZX12 seeds at different maturity stages.

To study whether the late-senescent germplasm can continue to synthesize assimilates while maintaining its green seed coat, we investigated character indexes relating to the yield and quality of the two germplasms for two consecutive years. Pod moisture content analysis revealed ZX12 to have significantly higher moisture than ZX4 beginning at 40 DAF; moreover, at 50 DAF, ZX12 reached values more than 10-fold of those observed in ZX4 (Figure S1A). At 45 DAF, the seed moisture content of ZX12 was also significantly higher than that of ZX4 (Figure S1B). In terms of the dry weight of 100 seeds, ZX4 showed a trend of first rising and then declining; there was no significant change between 30 DAF and 45 DAF, and the decline began at 50 DAF. Meanwhile, ZX12 showed a gradually rising trend, reaching the maximum weight value at 50 DAF (Figure S1C). The starch content analysis found that starch in the seeds of both germplasms decreased with increasing DAF, and the starch content of ZX4 was significantly lower than that of ZX12 at 50 DAF (Figure S1D). Meanwhile, soluble sugar analysis found ZX4 to have significantly higher TSS than ZX12 at 30 DAF; the sugar content of ZX4 then decreased, and later increased dramatically at 50 DAF. Meanwhile, the soluble sugar content of ZX12 was higher than that of ZX4 at 35 DAF, but lower at other observation time points. Ultimately, the difference was most significant at 50 DAF, when the soluble sugar content of ZX4 was more than twice that of ZX12 (Figure S1E). Finally, antioxidant enzyme activity assays revealed the SOD, POD, and T-AOC activities of ZX4 seeds to be significantly higher than those of ZX12 from 20 DAF (Figure 2).

### 3.3. Transcriptome Analysis of ZX4 and ZX12 Seeds at Different Stages of Maturity Revealed Differential Expression of Chl Metabolism and Photosynthesis Pathway-Related Genes

**Figure 3:**
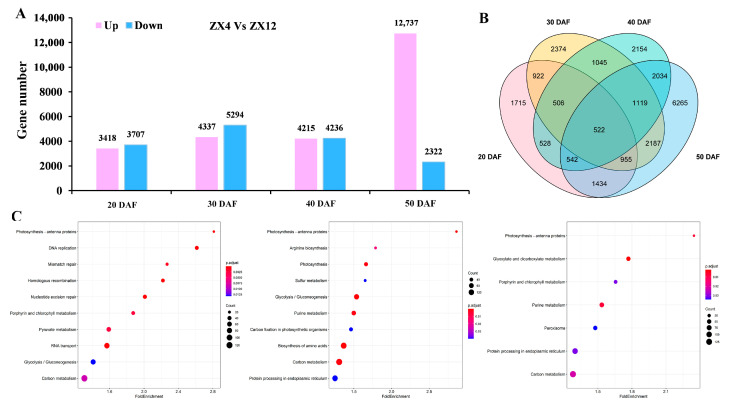
Transcriptome analysis of ZX12 and ZX4 seeds at different maturity stages. (**A**) Number of DEGs in ZX12 and ZX4 seeds at different maturity stages. (**B**) Venn diagram of DEGs between ZX12 and ZX4 seeds at different maturity stages. (**C**) KEGG analysis on the differentially expressed genes at 20, 30, and 40 DAF.

Eight cDNA libraries were constructed for ZX4 and ZX12 seeds collected at 20, 30, 40, and 50 DAF, and transcriptome analysis identified a total of 7125, 9631, 8451, and 15,059 DEGs for those respective time points (Figure 3A, Table S2). Overall, a set of 23,802 non-redundant DEGs was obtained, inclusive of all time points (Figure 3B). GO enrichment analysis was performed on the DEGs obtained for each time point (Figure S2). The most significantly enriched terms in each sub-ontology were: for biological process, metabolic process and cellular process; for cellular component, cell and intracellular; and for molecular function, catalytic activity and binding function (Figure 3C). 

We further performed KEGG enrichment analysis on DEGs to gain insight into the key regulators of Chl degradation during senescence. Given the almost complete degradation of Chl in ZX4 seeds at 50 DAF (Figure 1B), we analyzed only those genes differentially expressed at 20, 30, and 40 DAF (Figure 3C). Notably, this analysis found genes related to the Chl metabolism pathway or photosynthesis pathway to be enriched in each of the three periods. Chl metabolism is essential for maintaining plant photosynthesis and the green phenotype. Specifically, 52, 75, and 51 Chl metabolic pathway genes were enriched at 20, 30, and 40 DAF, respectively, and photosynthesis-antenna pathway genes were enriched at all time points.

**Figure 4:**
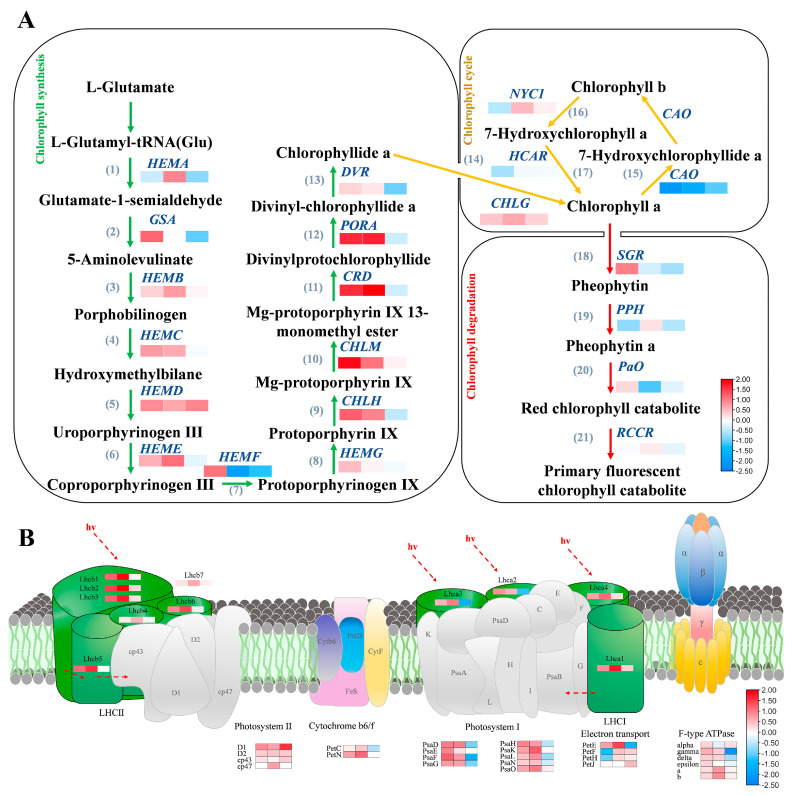
Chl metabolism and photosynthesis pathway-related genes are differentially expressed in ZX12/ZX4 at 20, 30 and 40 DAF time. (**A**) Heat map of Chl metabolism gene expression. (**B**) Heat map of key gene expression in photosynthesis pathway. The value of the heat map is the log_2_ (ZX12/ZX4) value of the ratio of the expression in RNA-seq.

We further analyzed the relative expression of key factors in Chl metabolism pathways at 20, 30, and 40 DAF (Figure 4A). At 20 and 30 DAF, ZX12 exhibited up-regulation of 11 key regulators of Chl synthesis (*GmGSA*, *GmHEMB*, *GmHEMC*, *GmHEMD*, *GmHEME*, *GmHEMG*, *GmCHLH*, *GmCHLM*, *GmCRD*, *GmPORA*, and *GmDVR*), of which *GmHEMA* was up-regulated relative to ZX4 at 20 DAF and *GmHEMF* down-regulated at 30 DAF. At 40 DAF, ZX12 exhibited higher expression levels of *GmHEMB*, *GmHEMC*, *GmHEMD*, *GmCHLG*, and *GmCHLM* than ZX4, and conversely lower levels of *GmHEMA*, *GmGSA*, *GmHEME*, *GmHEMF*, *GmHEMG*, *GmCHLH*, *GmCRD*, *GmPORA*, and *GmDVR*. Regarding Chl cycle genes, expression of *GmNYC1* in ZX12 was down-regulated relative to ZX4 at 20 DAF, but up-regulated at 30 and 40 DAF. Meanwhile, the expression of *GmHCAR* and *GmCAO* in ZX12 was down-regulated and *GmCHLG* was up-regulated at all time points. Finally, of the genes involved in Chl degradation, expression of *GmSGR* and *GmPAO* was up-regulated in ZX12 at 20 DAF relative to ZX4 and down-regulated at 30 and 40 DAF. Meanwhile, expression of *GmPPH* and *GmRCCR* was down-regulated in ZX12 at 20 and 40 DAF, and up-regulated at 30 DAF. qRT-PCR was performed to verify the expression levels of key factors involved in Chl synthesis, cycling, and degradation at 20 and 40 DAF. The results were consistent with the transcriptome profiles described above (Figure S3).

We similarly examined the expression of key genes in the photosynthesis pathway. Regarding photosynthetic system I, all genes were up-regulated in ZX12 relative to ZX4 at 20 and 30 DAF (namely *GmPsaD*, *GmPsaE*, *GmPsaF*, *GmPsaG*, *GmPsaH*, *GmPsaK*, *GmPsaL*, *GmPsaN*, and *GmPsaO*), while most genes were down-regulated at 40 DAF (specifically *GmPsaD*, *GmPsaF*, *GmPsaG*, *GmPsaH*, *GmPsaL*, and *GmPsaN*). Meanwhile, of genes in photosystem II, GmPetC was comparatively down-regulated in ZX12 at 40 DAF, but all other genes were up-regulated at all time points (namely *GmD1*, *GmD2*, *Gmcp43*, *Gmcp47*, and *GmPetN*). Several key genes involved in ATP synthesis were also mainly up-regulated in ZX12, namely genes of the Cytochrome b6f complex (*GmPetC* and *GmPetN*), F-type ATPase (*Gmalpha*, *Gmgamma*, *Gmdelta*, *Gmepsilon Gma*, and *Gmb*), and electron transport (*GmPetE*, *GmPetF*, *GmPetH* and *GmpetJ*). Genes down-regulated include *GmPetC*, *GmPetE*, *Gmgamma*, and *Gmdelta* at 40 DAF, and *GmPetF* and *GmPetH* at 20 DAF (Figure 4B).

### 3.4. Metabolome Analysis of ZX4 and ZX12 Seeds at Different Stages of Maturity Identified a Differential Abundance of Flavonoids

**Figure 5:**
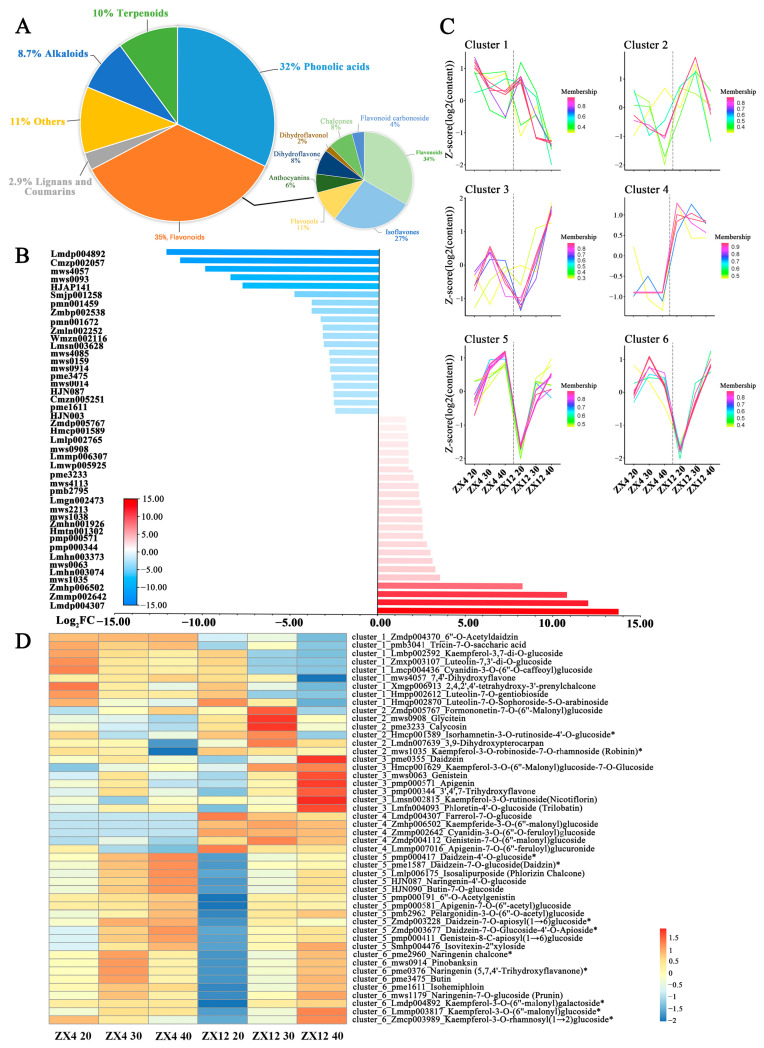
Metabolome analysis of differential metabolic profile between ZX4 and ZX12 seeds at different maturity stages. (**A**) Classification of differential metabolites. (**B**) The combination of the top 10 upregulating and downregulating metabolites in three periods. (**C**) Cluster analysis of expression patterns of differential metabolites. (**D**) Group metabolite expression heat map.

As the end product of the regulation of the life process, metabolite contents reflect levels of gene transcription and protein expression. In addition, the accumulation of metabolites determines a plant’s nutritional quality phenotype. We performed widely untargeted metabolic analysis on ZX4 and ZX12 soybean seeds at 20, 30, and 40 DAF to dissect the late-senescent mechanism at the metabolic level. A total of 294 metabolites were annotated in all samples, including flavonoids, alkaloids, terpenoids, phenolic acids, lignans, and coumarins. Metabolites with VIP scores ≥ 1 and Log_2_FC (fold change) ≥ 1 were considered to be differentially abundant in the two varieties. Overall, 56, 73, and 80 differential metabolites were identified at 20, 30, and 40 DAF, respectively. In total, 137 metabolites were differentially abundant in at least one stage, with 35% of those being flavonoids (Figure 5A). In particular, farrerol-7-O-glucoside (PubChem CID: 102255414, Log_2_FC ≥ 13.75), cyanidin-3-O-(6″-O-feruloyl) glucoside (Zmmp002642, Log_2_FC ≥ 12.01), and kaempferide-3-O-(6″-malonyl) glucoside (Zmhp006502, Log_2_FC ≥ 10.79) were specifically accumulated in ZX12 while absent in ZX4 at all three stages; this indicates that flavonoids are the primary contributors to the metabolic variation between ZX4 and ZX12 seeds (Figure 5B).

We next clustered the accumulation patterns of differentially abundant flavonoids at the three stages using C-Means clustering, which yielded six clusters (Figure 5C,D). The flavonoids in clusters 3, 4, and 6 had reduced or relatively low abundance in ZX4 while displaying increased or higher accumulation over time in ZX12. Meanwhile, flavonoids in clusters 1, 2, and 5 lacked any apparent patterns of differential accumulation between ZX4 and ZX12. We further mapped the differential flavonoids of clusters 3, 4, and 6 to the flavonoid, isoflavonoid, flavone, and flavonol biosynthesis pathways obtained from the KEGG database, which revealed them to be widely distributed at upstream or arterial locations in the biosynthesis pathway. For example, naringenin chalcone (PubChem CID: 5280960) and its product naringenin (PubChem CID: 932) in cluster 6 are key synthetic precursors of a series of downstream flavonoids. Farrerol (PubChem CID: 91144) is a downstream dimethyl derivative of naringenin; its glycosylation derivative, farrerol-7-O-glucoside, was a member of cluster 4 and was only detected in ZX12. Daidzein (PubChem CID: 5281708) and genistein (PubChem CID: 5280961) are the main aglycones of isoflavonoids, were members of cluster 3. Kaempferol (PubChem CID: 5280863) is a vital member of the flavonols and possesses antioxidant activity, thus can reduce the damage caused by oxidative stress. Its five glycosylation or malonyl glycosylation derivatives were included in clusters 3 and 6. Kaempferide (PubChem CID: 5281666) is a methyl derivative of kaempferol, and its malonyl glycosylation derivative (kaempferide-3-O-(6″-malonyl) glucoside) was only detected in ZX12. Taken together, the above results indicate that a relatively high level of flavonoid biosynthesis is maintained in ZX12 as its seed development proceeds to the mature stage.

### 3.5. Correlation Analysis of Differential Metabolites and DEGs in the Flavonoid and Anthocyanin Pathways

**Figure 6:**
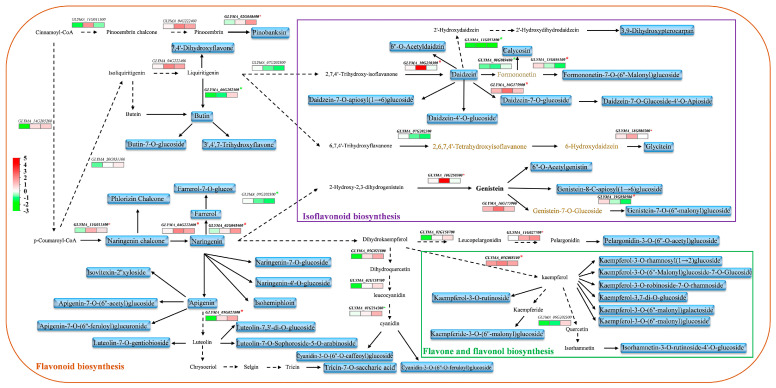
Correlation analysis between differential metabolites and differentially expressed genes of flavonoids and anthocyanins. Black color represents undetected metabolites; Purple represents detected metabolites; and the red asterisk represents a positive correlation and green represents a negative correlation.

We further analyzed the correlation between metabolite levels and the expression of key flavonoid and anthocyanin synthesis genes at the time points of 20, 30, and 40 DAF (Figure 6). The results showed that most of the detected flavonoids, isoflavonoids, and flavonol metabolite content were positively correlated with the expression level of synthetic genes, especially some of the metabolites at upstream or arterial locations in the synthetic pathway mentioned above, such as nalingenin chalcone (*GLYMA_04G222400*), Daidzein (*GLYMA_10G250300*), and kaempferol (*GLYMA_05G088100*). Farrerol-7-O-glucoside, which is higher in ZX12, is a derivative of Farrerol, and the content of Farrerol was also positively correlated with the synthetic gene *GLYMA_02G048400*. Downstream products kaempferide-3-O-(6″-malonyl) glucoside and cyanidin-3-O-(6″-O-feruloyl) glucoside were synthesized in large quantities in ZX12; however, the upstream hub products were all naringenin, and the genes involved in intermediate synthesis steps (*GLYMA_04G222400* and *GLYMA_05G088100*) exhibited a positive correlation.

## 4. Discussion

### 4.1. Unique Delayed Senescence Characteristics Suggest a New Regulatory Mechanism

For typical monocarpic plants, such as soybeans, leaf and even whole-plant senescence, and death are associated with the production of mature seeds, and the maintenance of photosynthetic capacity during the grain filling period is often related to the increase of grain yield [17,54,55]. In previous research, the initial process of delayed senescence in different crops was mainly described on leaves and delayed senescence in leaves has now become an agronomic desirable trait [56,57,58,59]. However, it has been found that continued photosynthesis of the pod wall is essential for grain filling, especially during plant senescence [60]. Although some cosmetic stay-green mutants have been found in soybeans, the seed coat and pod wall remain green after seed maturation. Still, the chloroplast has lost its photosynthetic capacity and cannot improve crop yield [33,36,39]. Accordingly, this study selected two soybean germplasms (ZX4 and ZX12) with stable cultivation traits and different delayed senescence characteristics for examination (Figure 1A). We first observed the seed late-senescent phenotype of the two germplasms at different DAF, and found ZX12 to have late-senescent characteristics when compared with ZX4; measurement of Chl content confirmed ZX12 to have higher Chl content from 20 DAF on. By measuring the Chl fluorescence intensity under different DAF, it was found that ZX12 had higher fluorescence intensity, suggesting that ZX12 had higher photosynthetic capacity in the seed maturation stage. In soybean, *GmSARK* (leucine-rich repeat receptor-like protein kinase) functionally deficient transgenic lines showed a late-senescent phenotype and higher Chl content than the wild type [61]. This was consistent with the phenotype of ZX12, but the correlation between its high chlorophyll content and crop yield is not described. The dry weight of 100 seeds of ZX12 was higher than that obtained for ZX4, indicating that the late-senescent phenotype of ZX12 is not due to interruption of the Chl degradation process (Figure S1C). The soybean delayed senescence mutant *z1* had a lower leaf Chl degradation rate and higher photosynthetic efficiency during maturation than the control varieties, consistent with the phenotype of ZX12 in this study [62]. For yield-related characteristics, although *z1* had a higher seed mass per plant, the 100-seed weight of *z1* was lower than that of control varieties, which was inconsistent with the result that ZX12 had a higher 100-grain dry weight than ZX4. The results suggest that tissue differences that delay senescence may affect crop yield. In addition, although ZX12 has a delayed senescence phenotype, the seed coat color of ZX12 still partially changes to yellow when the seed is fully mature. The study on the cosmetic stay-green soybean variety BN106 showed that the seed coat of *GmSGR*-deficient transgenic plants remained green when the seeds were fully mature [44]. The above results indicate that ZX12 was different from typical soybean cosmetic stay-green mutants. The subtle changes in its late-senescent phenotype may be due to a regulation mechanism different from those reported in previous studies.

### 4.2. Genes Relating to Chl Metabolism and Photosynthesis Are Involved in Regulating the Late-Senescent Phenotype

Transcriptome sequencing was performed on seeds from the two soybean varieties collected at different DAF. KEGG enrichment analysis of genes differentially expressed at 20, 30, and 40 DAF revealed enrichments of Chl metabolism and photosynthesis pathway-related genes. We further examined the expression of genes encoding key enzymes in the Chl metabolism pathway and found ZX12 to have significantly higher expression of Chl synthase; in addition, genes for Chl-degrading enzymes were partially up-regulated at the early stage of seed maturity. The significant upregulation of Chl synthase in ZX12 at 20 DAF was consistent with the higher Chl content in ZX12. It is also worth noting that genes encoding Chl-degrading enzymes were significantly down-regulated in ZX12 relative to ZX4 at 40 DAF. In tobacco and *cucumis melo*, overexpression of *CHLG* can activate the Chl synthesis pathway [63,64]. Meanwhile, interfering with *HEMA* mRNA expression in Arabidopsis can degrade Chl and accelerate the yellowing of leaves [65]. Overexpression of *PORA* in Arabidopsis *porB-1 porC-1* double mutants can increase Chl synthesis [66]. Therefore, it can be assumed that the high level of Chl synthesis in the early stage of seed maturation and the low level of degradation in the late stage of seed maturation contribute, at least in part, to the late-senescent phenotype of ZX12.

Regarding the photosynthesis system, Chl a/b antenna proteins are composed of photosystem (PS) I and II [67,68] while Light-harvesting complex (LHC) I and II combine with Chl a and Chl b to carry out photosynthesis [69]. Similar to Chl content, our analysis of the expression of key genes in the photosynthetic system showed that the system is mainly up-regulated in ZX12 at the time points examined. Previous studies have found the expression of photosynthetic genes to be affected by other factors (hormones, light intensity, carbon dioxide concentration, etc.) and negatively correlated with leaf senescence [67,70]. These results indicate ZX12 to have a higher photosynthetic efficiency than ZX4 during seed maturation, which is consistent with the continuous increase observed in the dry 100-seed weight of ZX12 through 50 DAF.

### 4.3. Flavonoids Inhibit Chlorophyll Degradation by Scavenging Oxygen Free Radicals

Plant metabolites play important roles in plant growth and development [71]. To study whether metabolite levels during seed maturation affect late-senescent phenotype of ZX12, we conducted an untargeted metabolic analysis of ZX4 and ZX12 seeds at 20, 30, and 40 DAF. In total, 294 metabolites were detected, of which flavonoids were the primary type to have differential abundances. Flavonoids are important compounds closely related to the color of plant organs. Recent studies have also found that flavonoids can regulate key enzymes, participate in antioxidant defense, have antimicrobial activity, and increase plant biological and abiotic stress resistance [72,73,74]. In particular, flavonoids can scavenge oxygen free radicals produced in plants, known as reactive oxygen species (ROS) [75]. These oxygen free radicals can induce oxidative damage in plants, leading to increased plant senescence and resistance [76]. Studies have shown that ROS accumulation and antioxidant enzymes correlate with chlorophyll content during senescence [77]. POD and SOD activities in mung beans were positively correlated with chlorophyll content [78]. The accumulation of ROS in cucumbers can regulate the expression of Chl catabolic enzymes and participate in the chlorophyll degradation process [18]. In *Arabidopsis thaliana*, AtWRKY42 accelerated age-dependent leaf senescence by accumulating H_2_O_2_ [35]. Therefore, flavonoids may constitute a ‘secondary’ antioxidant system activated due to the depletion of antioxidant enzyme activity. It was found that the antioxidant enzyme activity and Chl degradation rate of ZX12 were lower than that of ZX4, which was different from previous studies. It was speculated that the late-senescent germplasms were under lower stress or had other antioxidant components involved in scavenging ROS. In addition, compared with ZX4, ZX12 has three unique and abundant metabolites during senescence, all of which are flavonoids (farrerol-7-O-glucoside, cyanidin-3-O-(6″-O-feruloyl) glucoside, and kaempferide-3-O-(6″-malonyl) glucoside); accordingly, we speculate that flavonoids are the main protective substances in the late-senescent seeds of ZX12. It is worth noting that chloroplasts harbor “antioxidant” flavonoids (dihydroxy-B-ring-substituted flavonoids), which act as scavengers of singlet oxygen and stabilizers of the outer chloroplast membrane [79]. The flavonoids in chloroplasts may also maintain the integrity of the envelope through lipid remodeling during cell dehydration, thereby preventing oxidative damage [79,80,81,82]. Interestingly, the cyanidin-3-O-(6″-O-feruloyl) glucoside detected in ZX12 has a dihydroxy-B-ring structure, and farrerol-7-O-glucoside is a downstream derivative of farrerol, which has a monohydroxy-B-ring structure (Figure S4). The presence of these structures suggests these flavonoids may play key roles in scavenging oxygen free radicals in chloroplasts.

## 5. Conclusions

**Figure 7:**
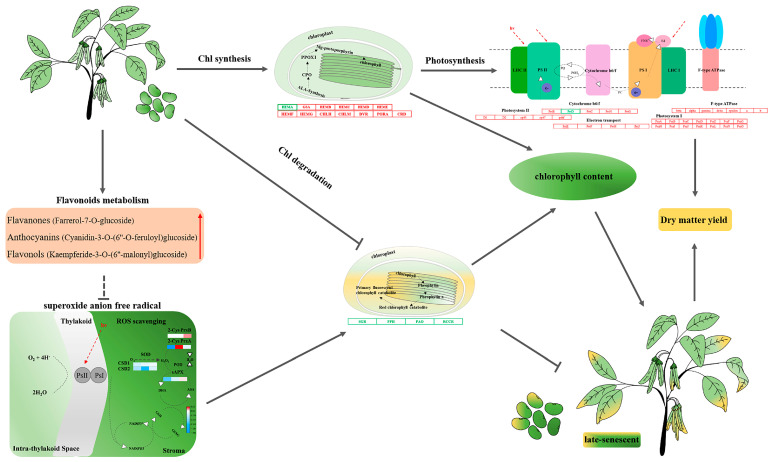
The proposed model affecting the difference of stay-green ability between ZX12 and ZX4 seeds at mature stages. The font in red indicates that they are positively regulated in ZX12; red boxes represent up-regulation; green boxes represent down-regulation; and the arrow represents promotion, and the T represents inhibition.

Based on the above results, we propose a hypothetical model to illustrate the regulation mode of ZX12 seed late-senescent phenotype (Figure 7). Relative to ZX4, ZX12 seeds exhibited high expression of Chl synthesis genes and maintained a high Chl synthesis rate in the early stage of senescence, and also featured low expression of Chl degradation genes and maintained a low Chl degradation rate in the late stage. The high expression of photosynthesis-related genes was consistent with the phenotype of seed photosynthesis in the later stage of maturity. In addition, the metabolomic analysis found flavonoids to be the main differentially expressed metabolites. Three flavonoid metabolites unique to ZX12 (farrerol-7-O-glucoside, cyanidin-3-O-(6″-O-ferroyl) glucoside, and kaempferide-3-O-(6″-malonyl) glucoside) may act to slow the degradation of Chl by scavenging oxygen free radicals in the chloroplasts. However, the specific regulation mechanism remains to be further studied.

## Data Availability

The sequence files presented in this study are openly available in NCBI, accession number: PRJNA904140. The metabolomics data presented in this study are openly available in The National Omics Data Encyclopedia, project ID: OEP003774.

## Supplementary Materials

The following supporting information can be downloaded at: https://www.mdpi.com/article/10.3390/antiox11122480/s1, Figure S1: Seed characters between ZX4 and ZX12 at different maturity stages; Figure S2: GO enrichment analysis of differential genes between ZX12 and ZX4; Figure S3: Expression level of Chl metabolism key genes validated by RT-qPCR in ZX12 and ZX4 seeds at 20 and 40 DAF time; Figure S4: Chemical structures of three flavonoid metabolites unique to ZX12; Table S1: Primers used for qRT-PCR; Table S2: Mapping statistics of RNA-Seq data.
