# Supplementary material for: Transcriptome and Metabolome Analysis of a Late-Senescent Vegetable Soybean during Seed Development Provides New Insights into Degradation of Chlorophyll
Source: Antioxidants (Basel). 2022 Dec 16;11(12):2480. doi: 10.3390/antiox11122480 (PMC9774520; doi:10.3390/antiox11122480)
Supplement: Supplementary file 1 [file antioxidants-11-02480-s001.zip › supplementary Figures.pdf]

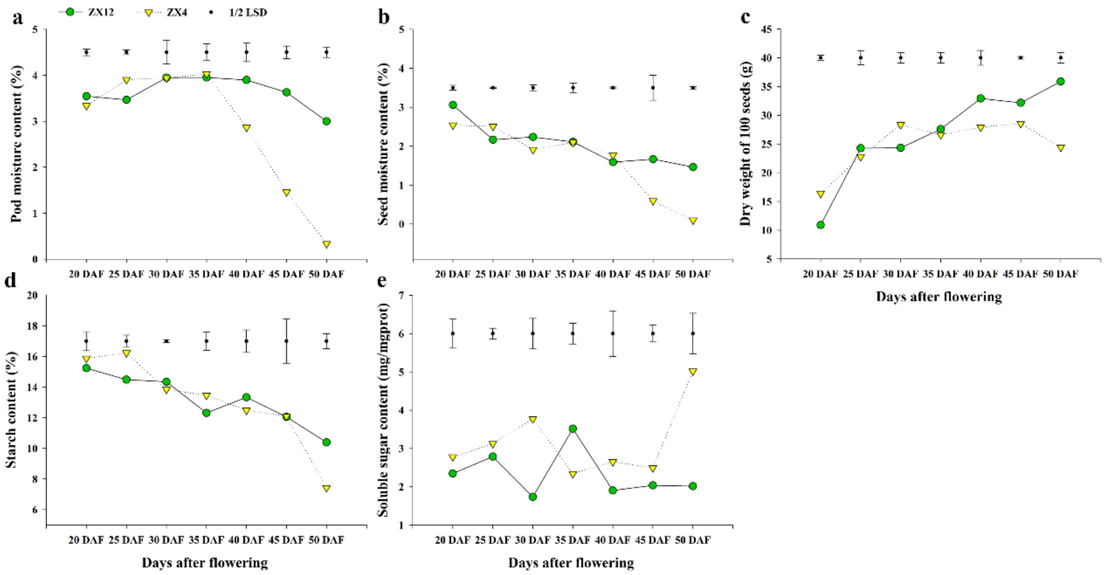

Fig S1 Seed characters between ZX4 and ZX12 at different maturity stages. a Moisture content of pods. b Moisture content of seeds. c Dry weight of 100 seeds. d Starch content of seeds. e Soluble sugar content of seeds.

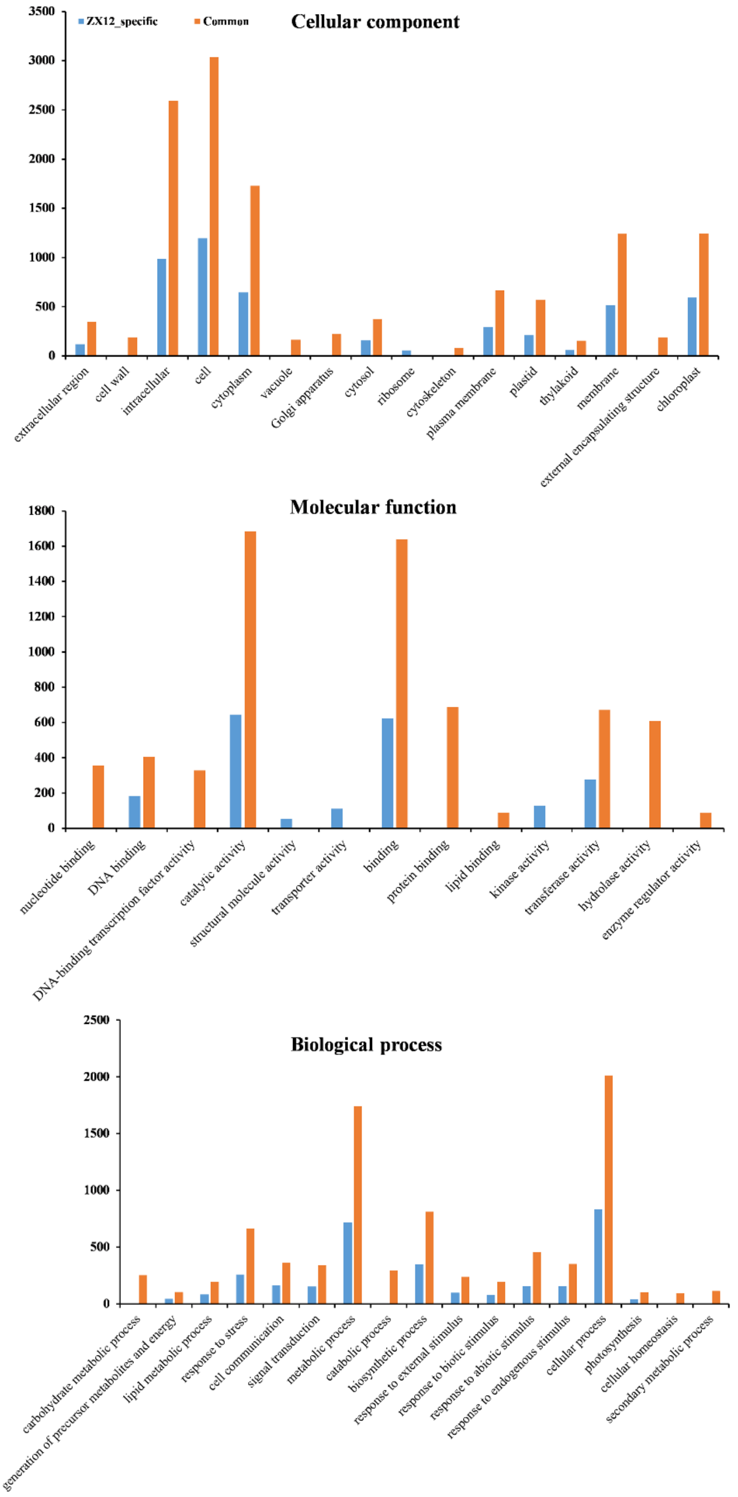

Fig S2 GO enrichment analysis of differential genes between ZX12 and ZX4

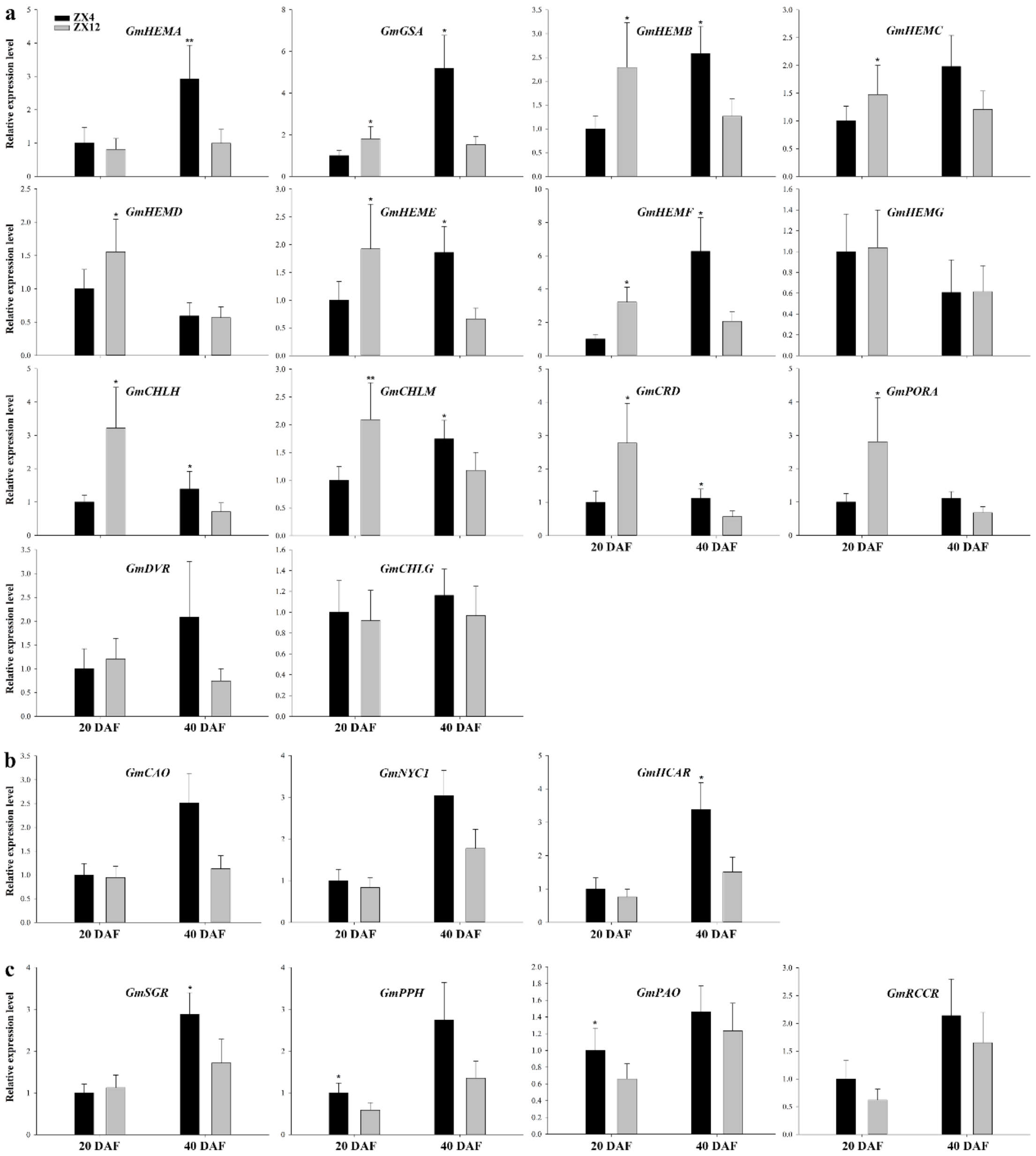

Fig S3 Expression level of Chl metabolism key genes validated by RT-qPCR in ZX12 and ZX4 seeds at 20 and 40 DAF time. a The expression level of Chl synthesis genes. b The expression level of Chl cycle genes. c The expression level of Chl degradation genes. Values represent the means ± SE of three biological replicates. Means with common letters are not significantly different at P≤0.05.

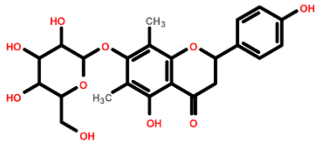

Farrerol-7-O-glucoside

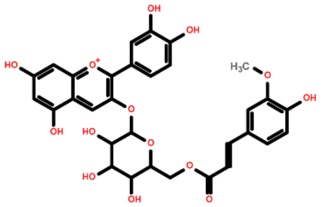

Cyanidin-feruloyl-glucoside

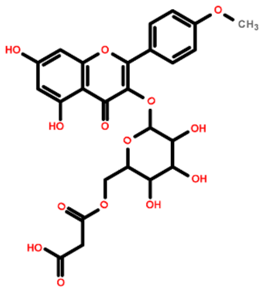

Kaempferide-malonyl-glucoside

Fig S4 Chemical structures of three flavonoid metabolites unique to ZX12
